# Trade and Health: Is the Health Community Ready for Action?

**DOI:** 10.1371/journal.pmed.0020008

**Published:** 2005-01-25

**Authors:** Kelley Lee, Meri Koivusalo

## Abstract

There are greater tensions than ever before between promoting trade and protecting health. Human health could lose out to trade liberalization unless the health community fights its case

Trade is the lifeblood of all commerce. The exchange of goods and services has played a defining role in human history, creating vast empires, encouraging mass migration, and sometimes tipping the balance between peace and conflict. It is thus unsurprising that protecting and encouraging international trade has remained a top priority for governments, businesses and international organisations.

Historically, the protection of health has been a permitted reason for restricting trade. Trade brought plague to Athens in 430 BC, killing as much as one third of the population, as well as to fourteenth century Europe after which quarantine practices were introduced. During the nineteenth century, flourishing trade also facilitated the spread of diseases such as cholera. This prompted a series of International Sanitary Conferences among leading trading nations, and the adoption of International Sanitary Conventions (forerunners of the present day International Health Regulations). While protecting health was a clear aim, in reality the primary task was to minimise interference by health matters on trade.

Today, there are greater tensions than ever before between promoting trade and protecting health because of globalisation. Successive rounds of trade negotiations held since the Second World War, under the General Agreement on Trade and Tariffs and, since 1995, the World Trade Organization (WTO), have substantially reduced tariff levels and standardised trading practices across countries. This process of trade liberalisation has significantly increased trade volumes, bringing more and more countries into the world trading system. For the public health community, trade has raced ahead of corresponding measures to protect health. Efforts to ensure that there is an appropriate balance between the two policy areas has become a difficult challenge.

## Tensions between Trade and Health

The right to restrict trade to protect the health of humans, animals, and plants is recognised by the General Agreement on Trade and Tariffs (Article XX) under two conditions: (1) the restriction is applied in a nondiscriminatory way; and (2) the restriction is based on recognised scientific evidence. Countries are allowed to restrict trade, for example, of certain goods such as radioactive waste or infected food products.

Disputes can arise if the restriction is believed to be discriminatory or there is disagreement about the scientific evidence supporting it (see sidebar). The ban introduced by the European Community in 1989 on hormone-treated beef imported from the US. led to two rulings by the Dispute Settlement Body of the WTO in favour of the American government. The assessment of the evidence primarily by trade experts, rather than public health experts, is a clear problem of the existing dispute settlement process. The process also makes it difficult to regulate inappropriate production methods which do not lead to problems in the end product but may be of public health concern. For example, the practice of using hormones to boost meat production may prove problematic in future research, even if residues in the meat are not judged high enough at present to warrant sufficient proof of health concerns.

Moreover, tight regulations on trade that are intended to protect health can come under fire from the trade lobby. Two World Bank studies argue, for instance, that European Union (EU) regulations on pesticides in bananas, as well as aflatoxins, could be interpreted as barriers to trade and market access [1,2].

The short shrift given to precautionary measures to protect health, where existing scientific evidence is deemed insufficient, reflects a further inbuilt priority given to trade. Growing concerns over the development and use of genetically modified organisms (GMOs), for example, have been dismissed by major companies such as Monsanto and Cargill on the basis of a lack of existing scientific evidence of harm to health. Consumer groups and public health advocates, however, argue that the subject is still in its scientific infancy. Where new causal pathways or systemic impacts of environmental exposures are of concern, such as with GMOs, at best one can say that the jury is still “out”. Hence, allowing GMOs to be spread widely, rather than taking precautionary measures, could prove to have irreversible consequences.[Fig pmed-0020008-g001]

**Figure pmed-0020008-g001:**
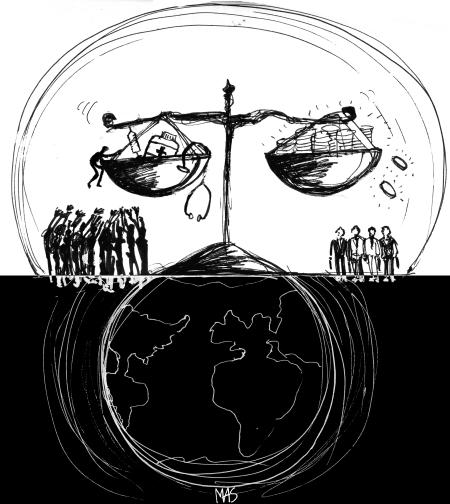
There is a fine balance between protecting patents and ensuring access to essential drugs in the developing world (Illustration: Margaret Shear)

The classification of certain goods as a risk to health, and thus subject to trade restrictions, is also a source of dispute. The best example is tobacco products which manufacturers argue should be treated like other traded goods. Public health advocates, however, argue that because tobacco is harmful to health, it should be subject to special restrictions. A battle over tobacco is currently being played out in regional trade negotiations and will be raised at forthcoming multilateral negotiations over agricultural trade. Whether the public health community will be able to argue successfully to protect health, when pitted against the vast resources of a multi-billion dollar industry, remains to be seen.

## The TRIPs Agreement

Two new sources of tension have arisen in recent years—intellectual property rights (IPRs) and trade in services. The protection of IPRs is a new feature of international trade law, coming into force under the Agreement on Trade-Related Aspects of Intellectual Property Rights (TRIPS) adopted in 1994. The agreement sets out minimum standards for protecting and enforcing patents, trademarks, and copyrights.

Since the mid-1990s there has been increasing concern over access to drugs in the developing world, and especially drugs for treating HIV/AIDS. This issue gained international attention when the South African government sought to access cheaper versions of patent protected drugs, but was faced with strong opposition by the pharmaceutical industry. The importance of public health priorities, and the existence of flexibility within TRIPS, was eventually confirmed in the Doha Declaration on the TRIPS Agreement and Public Health signed in 2001 [3]. The agreement allows for “compulsory licensing”, which means that local manufacturers in poor countries are allowed to make cheap versions of patented drugs during public health emergencies, provided that they give a royalty payment to the patent holder. Nonetheless, IPRs protection has remained a problem given continued disagreement by the US over which diseases and countries are covered under the declaration. The capacity to protect IPRs has also been enhanced by the shift towards bilateral trade negotiations as a result of the breakdown in multilateral negotiations under the WTO.

While international debates over TRIPS have so far focused on the developing world, it is clear that there are issues relevant to the public health community as a whole concerning open access to a wide range of health-related knowledge and information. The possible benefits and costs of the TRIPS agreement had not been openly discussed beforehand. Rather, its measures were heavily influenced by industries seeking to exert ownership over intellectual goods such as research, information, and other data sources. Within an increasingly competitive world market, companies are driven to recoup their investment in such resources through IPRs. For the public health community, however, there is a vital need for affordable and open access by all to such resources. Leaving the commercial market to drive research and development (R&D) can lead not only to problems of access in developing countries but it can also lead to the neglect of research and public health priorities in all countries, such as research on antibiotics, which has been deemed insufficiently profitable [4]. Concern over the impact of intellectual property rights and marketing monopolies on overall pharmaceutical policies, pricing and R&D of pharmaceuticals has also led to proposals for a new trade framework for global health care R&D efforts [5].

## Trade in Health Services

The General Agreement on Trade in Services (GATS) is another expanded area of trade law. Services are the fastest-growing segment of the world economy, providing more than sixty percent of global output and employment. In the past, most services were not considered to be tradable across borders. Advances in communications technology, including the rise of e-commerce, and regulatory changes have made it easier to deliver services across borders. For trade in health services, the implications are not yet clear.

It is generally expected that GATS would not apply to public services due to a general exemption on the matter. However, this exclusion is very narrowly worded, and based on a model of public services which may no longer hold. Health sector reforms have changed the ways in which publicly financed services are provided in many countries, including the extensive use of contracting out and managed competition. In most countries, this includes both medical care and related services such as laboratory or ambulance services. Health services, which are contracted out to the non-profit or for-profit sector, are likely to be no longer considered purely “public” and thus protected by the exclusion. Recent legal reviews of the GATS [6,7] confirm this concern, describing how the agreement would apply to any health-related service supplied on a “commercial” basis. Whether paid for directly by the patient or through a social security fund, the expectation that GATS negotiations will not apply to public services may no longer hold.

Given this, the two reviews warn that there are valid grounds to suggest that negotiations on trade in services, and full commitments in health services in GATS, will have important implications for the ways in which national health systems and policies are implemented. While GATS may not directly limit the aims of national health policies, commitments under GATS can influence the ability of governments to implement health policies and regulate commercial service providers. This could apply especially to efforts to introduce new regulations that restrict market forces. The current GATS negotiations on domestic regulation include requirements of least trade restrictiveness and necessity tests for introducing regulatory measures in committed sectors. These requirements could pose difficulties if a government sought, for example, to oblige hospitals to operate on a non-profit basis. This might be interpreted under the GATS as restricting market forces. In this way, GATS could effectively influence the scope of national health policy, even challenging the capacity of governments to pursue health policies that prioritise universal access, cost containment, and quality control.

## Challenges to the Health Community

As world trade continues to expand in scale and scope, the health community faces a number of key challenges. We must be armed with a better understanding of the world trading system, notably the legal framework for international trade. Comprehending the potential health implications of various bilateral, regional, and multilateral agreements regulating trade today is a daunting task. Although the public health community has fewer resources at its disposal than the proponents of trade liberalisation, it is clear that health priorities do have strong support by citizens all over the world.

It is critically important for the health community to challenge the value-based assumption that trade liberalisation, rather than human welfare, should be given automatic priority. Indeed, ignoring health can lead to problems in the trade sphere. The outbreaks of bovine spongiform encephalopathy in Europe and North America and severe acute respiratory syndrome in Asia emphasised that trade can be severely disrupted by insufficient measures to protect health. The health community has to press for a much louder voice in the setting of trade policy at the national and international levels. A balance between trade and health policies can only be achieved if the health community is prepared to be far more vocal, and the trade policy community is prepared to listen.

**Recent Disputes between Trade and Health**

**Growth-Promoting Hormones**

In 1998 WTO ruled that a European Community ban of the use of certain growth-promoting hormones was not based on an appropriate risk assessment. Rather than lift the ban, the EU sought new evidence of the risk to human health of hormone residues in meat products. In 1999 Canada and the US imposed trade sanctions worth US$116.8 million and Cdn$11.3 million. In 2003 the EU announced that, based on new scientific evidence, the ban will remain in place and asked the US and Canada to lift sanctions.

**Chrysotile Asbestos**

In 2001 WTO ruled that the European Community was permitted to ban the import of chrysotile asbestos on the grounds of protecting public health, and rejected the Canadian government's claim that the ban was discriminatory and an unnecessary barrier to trade. While the decision shows that WTO panel decisions can prioritise health, the process and grounds of the decision indicates that, even with such a well-established carcinogen, the dispute settlement process required extensive argument by the public health community.

## Abbreviations

EU: European Union
GATS: General Agreement on Trade in Services
GMOs: genetically modified organisms
IPRs: intellectual property rights
R&D: research and development
TRIPS: Agreement on Trade-Related Aspects of Intellectual Property Rights
WTO: World Trade Organization
